# Supplementary figures and images for: Highly efficient maternal-fetal Zika virus transmission in pregnant rhesus macaques
Source: PLoS Pathog. 2017 May 25;13(5):e1006378. doi: 10.1371/journal.ppat.1006378 (PMC5444831; doi:10.1371/journal.ppat.1006378)

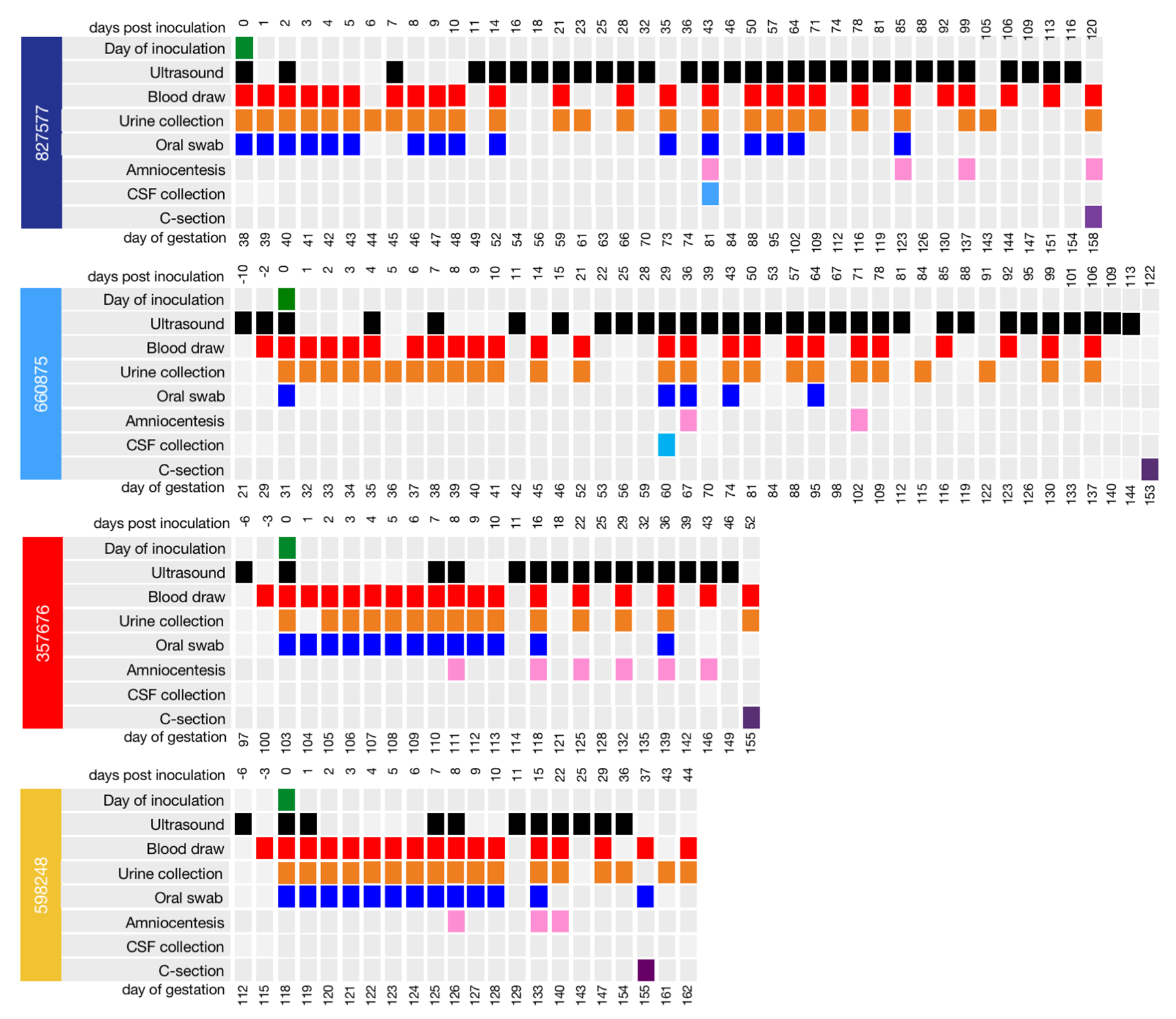

Supplement: S1 Fig — Each animal in the study is indicated at the left, color blocks represent when specific samples were collected (e.g., CSF on 43 dpi (81 days gestation) for animal 827577). (TIF) [file ppat.1006378.s001.tif]

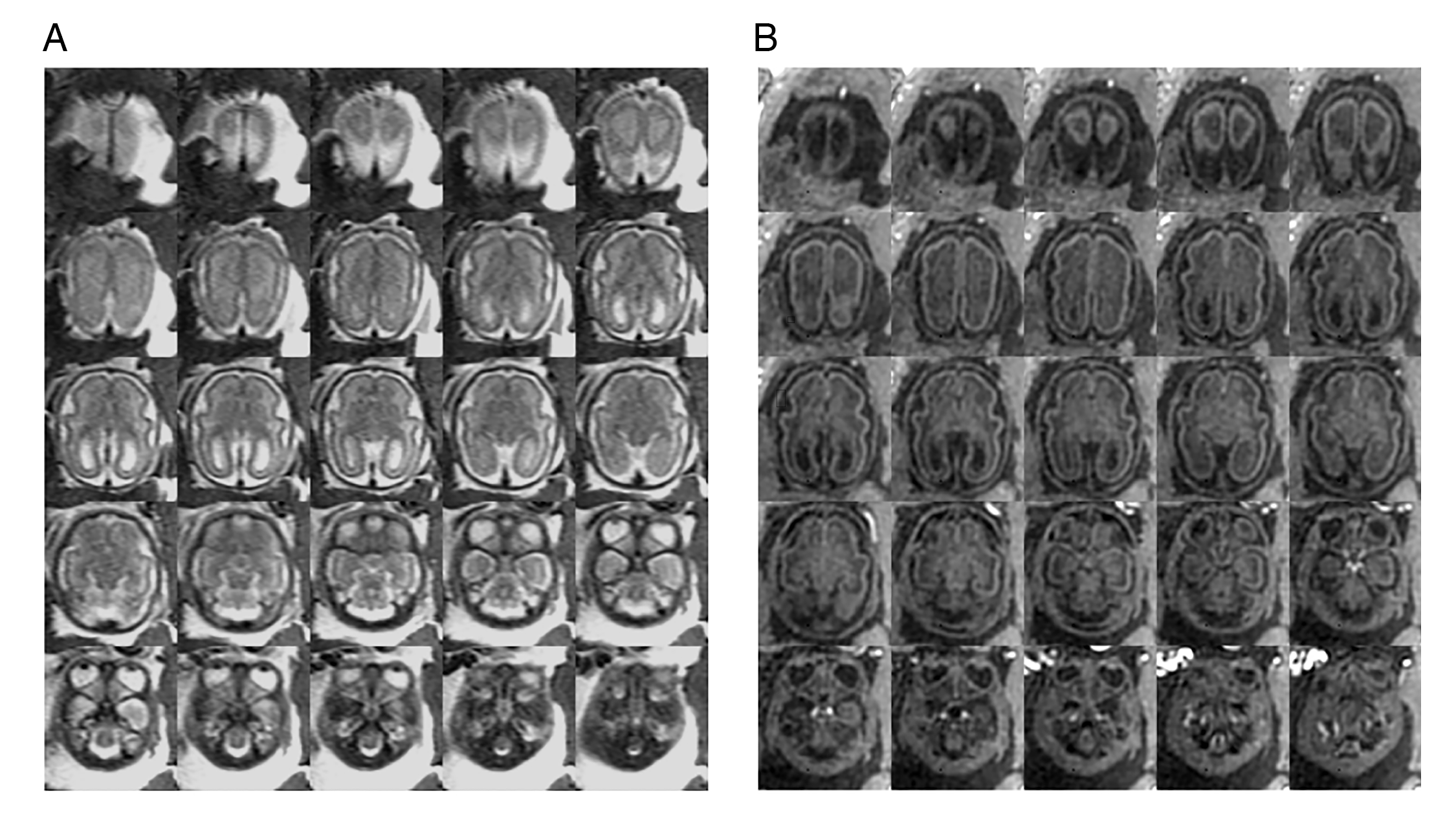

Supplement: S2 Fig — (A) T2-weighted axial images of the fetus from dam 660875 at 60 dpi (91 days gestation) acquired with a single shot fast spin echo (SSFSE) sequence. Fluids such as the intraocular fluid, CSF, and amniotic fluid as well as fat appear bright on these images. The brain anatomy appears normal. (B) The same fetus acquired with a multiecho spoiled gradient echo sequence. (TIF) [file ppat.1006378.s002.tif]

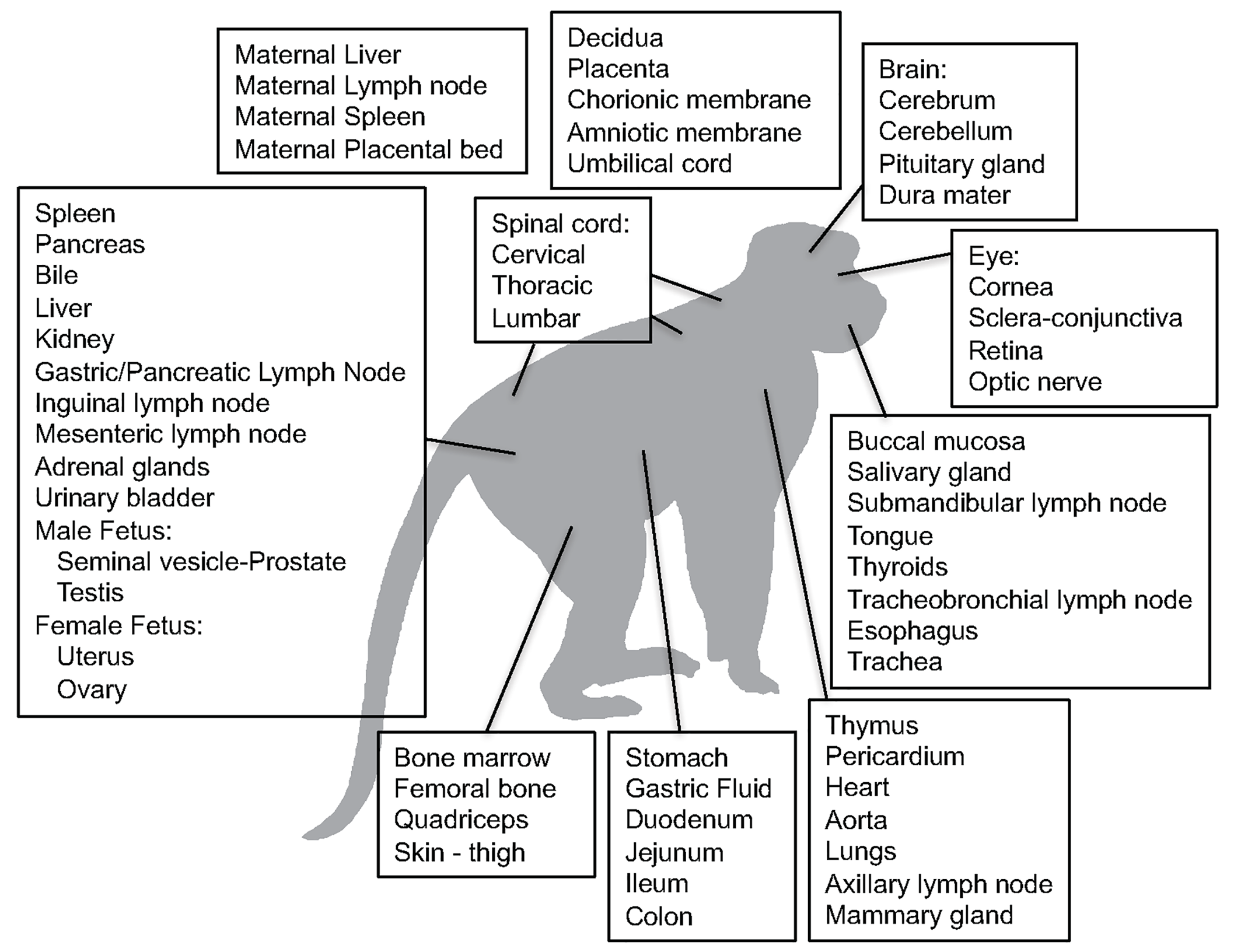

Supplement: S3 Fig — (TIF) [file ppat.1006378.s003.tif]
