# Supplementary material for: Highly efficient maternal-fetal Zika virus transmission in pregnant rhesus macaques
Source: PLoS Pathog. 2017 May 25;13(5):e1006378. doi: 10.1371/journal.ppat.1006378 (PMC5444831; doi:10.1371/journal.ppat.1006378)
Supplement: S1 Data — (PDF) [file ppat.1006378.s004.pdf]

Supplementary Data S7. Morphologic diagnoses from gross and histologic examination of maternal, fetal, and maternal-fetal interface tissues.

Necropsy 598248:
- Placenta: minimal to mild multifocal suppurative and necrotizing placentitis.
- Decidua: minimal lymphocytic and neutrophilic deciduitis.
- Amnion: mild diffuse suppurative amnionitis.
- Chorion: Mild diffuse suppurative chorioamnionitits with multifocal mineralization.
- Spleen, fetus: minimal diffuse splenitis.
- Pancreatic lymph node, fetus: moderate diffuse suppurative lymphadenitis.
- Gastric lymph node, fetus: mild diffuse suppurative lymphadenitis.
- Lungs, fetus: mild diffuse suppurative alveolitis (pneumonia) with intra-alveolar squamous cells and occasional macrophages.
- Liver, fetus: mild suppurative and lymphoplasmacytic cholangiohepatitis and serositis with mild multifocal extramedullary hematopoiesis.

Necropsy 357676:
- Placenta: moderate multifocal suppurative placentitis with thrombosis and multifocal mineralization.
- Decidua: mild multifocal suppurative deciduitis with thrombosis and multifocal mineralization.
- Amnion: Severe diffuse edema with minimal diffuse suppurative amnionitis.
- Chorion: moderate multifocal to diffuse suppurative chorioamnionitis.
- Spleen, maternal: mild suppurative splenitis.
- Axillary lymph node, fetus: minimal suppurative lymphadenitis.
- Inguinal lymph node, fetus: minimal suppurative lymphadenitis.
- Lung, fetus: scattered single intra-alveolar squamous cells and minimal pulmonary edema.
- Pericardium, fetus: mild multifocal nodular lymphoid hyperplasia.
- Liver, fetus: minimal multifocal extramedullary hematopoiesis.
- Buccal mucosa, fetus: mild multifocal ballooning degeneration.

Necropsy 827577:
- Placenta: moderate multifocal suppurative placentitis, multifocal mineralization, and perivascular lymphocytic infiltration.
- Decidua: moderate multifocal suppurative deciduitis.
- Amnion: minimal multifocal suppurative amnionitis, multifocal perivascular lymphocytic infiltration.
- Spleen, maternal: mild diffuse suppurative splenitis.
- Eye, fetus: Minimal lymphocytic iritis, minimal lymphocytic conjunctivitis, mild peripheral retinal and optic nerve gliosis.
- Spleen, fetus: Mild suppurative splenitis.
- Lungs, fetus: moderate intra-alveolar squamous cells.

Necropsy 660875
- Placenta: Moderate multifocal necrosuppurative placentitis.
- Decidua: mild to moderate diffuse suppurative deciduitis.
- Spleen, maternal: moderate diffuse suppurative splenitis with severe multifocal lymphoid hyperplasia with follicular dysplasia.

- Liver, maternal: minimal multifocal periportal lymphosuppurative hepatitis.
- Lymph node, maternal: moderate diffuse lymphoid hyperplasia.
- Umbilical cord, infant: focal fibrin thrombus with intralesional neutrophils.
- Eye, fetus: minimal lymphocytic iritis, moderate neutrophilic cyclitis, minimal lymphocytic conjunctivitis, moderate optic nerve gliosis.
- Spleen, fetus: mild diffuse suppurative splenitis.
- Axillary lymph node, fetus: moderate diffuse suppurative lymphadenitis.
- Submandibular lymph node, fetus: moderate diffuse suppurative lymphadenitis.
- Inguinal lymph node, fetus: moderate diffuse suppurative lymphadenitis.
- Lungs, fetus: Moderate diffuse suppurative alveolitis with intra-alveolar squamous cells.
- Liver, fetus: minimal multifocal suppurative periportal and serosal hepatitis.
